# The Microbiome of *Posidonia oceanica* Seagrass Leaves Can Be Dominated by Planctomycetes

**DOI:** 10.3389/fmicb.2020.01458

**Published:** 2020-07-10

**Authors:** Timo Kohn, Patrick Rast, Nicolai Kallscheuer, Sandra Wiegand, Christian Boedeker, Mike S. M. Jetten, Olga Jeske, John Vollmers, Anne-Kristin Kaster, Manfred Rohde, Mareike Jogler, Christian Jogler

**Affiliations:** ^1^Department of Microbiology, Radboud University, Nijmegen, Netherlands; ^2^Leibniz-Institut Deutsche Sammlung von Mikroorganismen und Zellkulturen, Braunschweig, Germany; ^3^Institute for Biological Interfaces 5, Karlsruhe Institute of Technology, Eggenstein-Leopoldshafen, Germany; ^4^Central Facility for Microscopy, Helmholtz Centre for Infection Research, Braunschweig, Germany; ^5^Department of Microbial Interactions, Institute of Microbiology, Friedrich Schiller University, Jena, Germany

**Keywords:** planctomycetes, seagrass, microbiome, taxonomy, biofilm

## Abstract

Seagrass meadows are ubiquitous, fragile and endangered marine habitats, which serve as fish breeding grounds, stabilize ocean floor substrates, retain nutrients and serve as important carbon sinks, counteracting climate change. In the Mediterranean Sea, seagrass meadows are mostly formed by the slow-growing endemic plant *Posidonia oceanica* (Neptune grass), which is endangered by global warming and recreational motorboating. Despite its importance, surprisingly little is known about the leaf surface microbiome of *P. oceanica*. Using amplicon sequencing, we here show that species belonging to the phylum *Planctomycetes* can dominate the biofilms of young and aged *P. oceanica* leaves. Application of selective cultivation techniques allowed for the isolation of two novel planctomycetal strains belonging to two yet uncharacterized genera.

## Introduction

**FIGURE 1:**
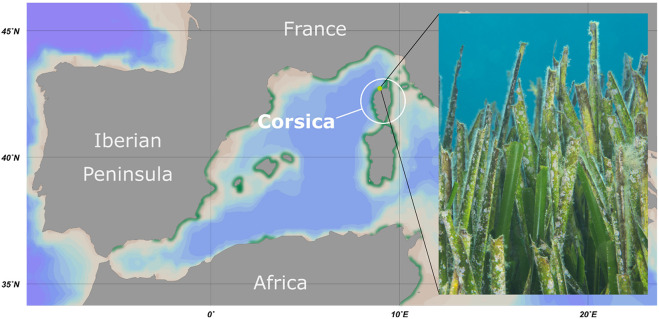
Overview of the sampling location. Map of the western Mediterranean Sea including the sampling area at the island Corsica. The distribution of seagrass meadows is depicted in green. The sampling point at STARESO diving station is marked in light green. The photograph shows a typical section of a *Posidonia oceanica* seagrass meadow of the sampling area.

Seagrasses are a paraphyletic group of angiosperm (higher) plants, which are exclusively found in estuarine and marine environments (Larkum et al., 2006). They belong to four families, *Posidoniaceae*, *Zosteraceae*, *Cymodoceaceae*, and *Hydrocharitaceae* (den Hartog and Kuo, 2006). Among these, the endemic species *Posidonia oceanica* is predominant in the Mediterranean Sea (Papenbrock, 2012) (Figure 1). *Posidonia* meadows provide breeding and nursery grounds for various fish and other marine organisms. They influence commercial fishing and shape the coastal structure by accumulating nutrients. *Posidonia* meadows are primary biomass producers and hence important for global carbon cycling (Hofrichter, 2002). They serve as nutrient hotspots in the otherwise oligotrophic surrounding seawater (Hofrichter, 2002), while their capacity to assimilate carbon exceeds the potential of many terrestrial ecosystems, such as boreal forests (Laffoley and Grimsditch, 2009). *P. oceanica* meadows influence food webs from shallow bays to water depths down to 40 m. Strikingly, *P. oceanica* is a threatened species and decreasing populations contribute less and less to the global carbon sink (Pergent et al., 2016). Besides their role in carbon cycling, either *Posidonia* plants or their epiphytes are suggested to produce small molecules with potential application in human medicine (Heglmeier and Zidorn, 2010; Zidorn, 2016). Due to importance in the ecosystem of the Mediterranean Sea, *P. oceanica* has been thoroughly investigated. Seasonal growth patterns as well as evidence for the alarming decline rates due to habitat pollution and global warming-associated environmental changes have been reported (Waycott et al., 2009; Short et al., 2011; Pergent et al., 2015).

However, only few studies focused on microbial communities associated with *P. oceanica* (Novak, 1984). This is astonishing, given the mutualistic relationship of terrestrial plants and associated microorganisms. The latter cannot only promote growth, but also benefit their host in terms of salt stress tolerance (Vacheron et al., 2013; Ledger et al., 2016). The little research on the microbiome associated with *P. oceanica* mostly focused on the root system (Torta et al., 2015; Vohnik et al., 2015, 2016) and on bacterial endophytes (Garcias-Bonet et al., 2012). Hitherto, only very few studies focused on the seagrass leaf microbiome, for example of eelgrass (*Zostera marina*), a plant endemic in the coastal regions of the northern hemisphere (Bengtsson et al., 2017; Ettinger et al., 2017). These studies indicated high variability in the composition of microbial species and pointed to rather low abundances of members of the phylum *Planctomycetes* (Bengtsson et al., 2017; Ettinger et al., 2017). This is surprising, given that all sorts of surfaces in aquatic habitats, such as macroalgae (Bengtsson and Øvreås, 2010; Bengtsson et al., 2012; Bondoso et al., 2014, 2015; Lage and Bondoso, 2014), crustaceans (Kohn et al., 2016), plants (Yadav et al., 2018), marine snow (DeLong et al., 1993) and cyanobacterial aggregates (Cai et al., 2013), were shown to be heavily colonized by *Planctomycetes*. Members of this phylum occur ubiquitously and sometimes even represent the dominant entity in such biotic surface microbiomes. Values of up to 70% of the bacterial community composition were reported (Wiegand et al., 2018). Phylogenetically, the phylum *Planctomycetes*, together with *Verrucomicrobia, Chlamydiae*, *Lentisphaerae*, *Kiritimatiellaeota*, and *Candidatus* Omnitrophica, forms the PVC superphylum (Wagner and Horn, 2006). Species of the phylum feature an extraordinary cell biology among bacteria (Jeske et al., 2015; Kohn et al., 2016; Boedeker et al., 2017; Wiegand et al., 2018). For example, they mostly divide by polar budding and lack otherwise universal bacterial division proteins, such as FtsZ (Jogler et al., 2012; Wiegand et al., 2020). Several representatives of the order *Planctomycetales* feature a biphasic life cycle, switching between a planktonic flagellated swimmer and a sessile reproduction stage (Franzmann and Skerman, 1984; Gade et al., 2005; Jogler et al., 2011; Jogler and Jogler, 2013). The periplasmic space of Planctomycetes is often enlarged and probably used for the degradation of polymeric compounds (Boedeker et al., 2017). Most Planctomycetes are rather slow-growing bacteria, especially compared to other heterotrophic bacteria occurring in the same ecological niches (Wiegand et al., 2018). Consequently, the dominance of Planctomycetes in competitive habitats, such as nutrient hotspots in the otherwise oligotrophic ocean appears enigmatic. One explanation for this phenomenon may be production of small bioactive molecules by the colonizing Planctomycetes to defend their ecological niche by means of ‘chemical warfare.’ The notion that Planctomycetes might be ‘talented producers’ of such small molecules is also supported by the large genomes of up to 12.4 Mb (Ravin et al., 2018) and predicted gene clusters for secondary metabolite production (Jeske et al., 2013, 2016; Graca et al., 2016; Wiegand et al., 2018, 2020).

In this study, we analyzed the bacterial community composition of *P. oceanica* leaves using electron microscopy, 16S rRNA gene amplicon sequencing and a cultivation-dependent approach targeting yet unknown members of the phylum *Planctomycetes*.

## Materials and Methods

### Sampling of Seagrass Material

**FIGURE 2:**
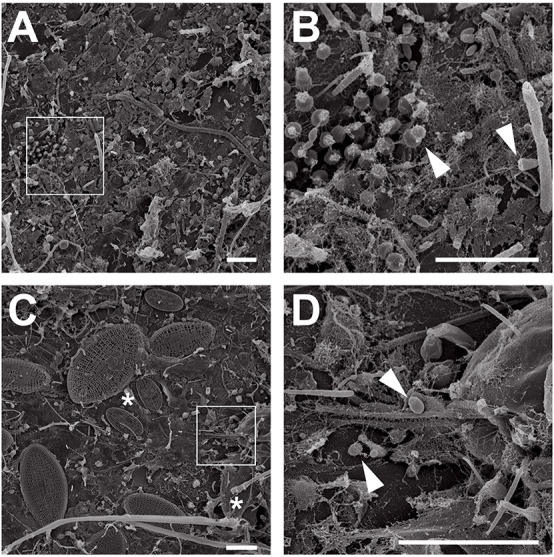
Electron microscopic analysis of seagrass biofilms. Scanning electron micrographs of microorganisms in young and aged *Posidonia oceanica* leaf biofilms. Overview images show the morphological differences between young **(A)** and aged leaf **(C)** epiphytic biofilm communities. While young leaves are colonized primarily with bacteria, aged leaves show a frequent colonization with diatoms (**C**, white asterisks). Areas indicated by white squares in **(A,C)** are shown at higher magnification in **(B,D)**. Cells showing planctomycete-like morphology on young **(B)** and aged **(D)** leaves are marked with arrows. Scale bar indicates 6 μm.

**FIGURE 3:**
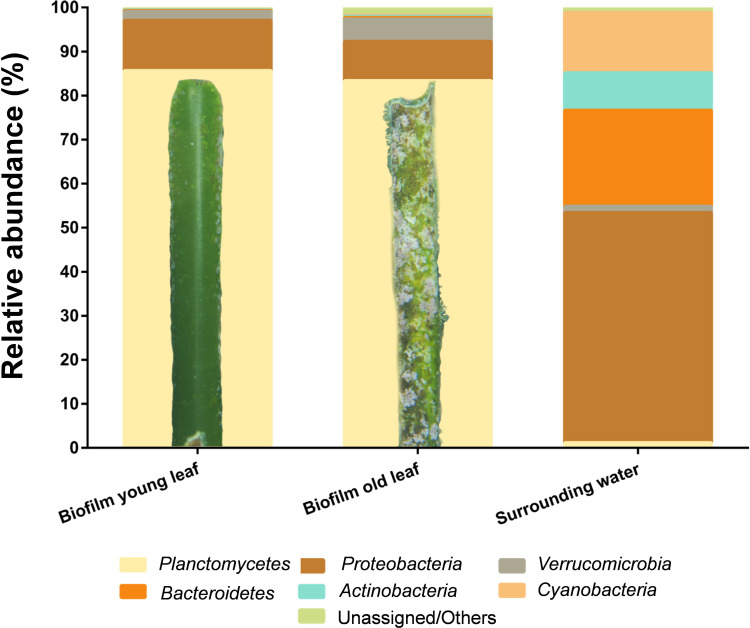
Cultivation-independent analysis of seagrass-associated bacterial communities. Amplicon-based bacterial community profiles of young and old *Posidonia oceanica* leaf biofilms and of the surrounding water. Both biofilms, on young and old leaves, are dominated by members of the phylum *Planctomycetes* (yellow) with >80%. A detailed photograph is shown for the young and old leaf showing macroscopic differences. The surrounding water yielded lower abundance of *Planctomycetes* (<2% of the community) and was dominated by members of the phylum *Proteobacteria* with >60% of the total community.

Young and aged *P. oceanica* leaves were sampled by scientific scuba divers near the coastal shoreline of Corsica, France (date: September 29, 2015; location: 42.579N 8.725 E). On young leaves, little to no macroscopic epiphytes were visible (less than 1% coverage of the leaf surface) and leaf shape was sharp (see Figures 1–3 for representative examples). In contrast, old leaves were heavily colonized by all sorts of organisms (more than 20% coverage of the leaf surface), while leaf shape was fringed (see Figures 1–3 for representative examples). In addition, surrounding water from 15 m depth was collected in triplicates using sterilized 1 L bottles. Samples were immediately transferred to the STARESO laboratories (Pointe Revelatta, Corsica) and processed within 8 h. Sample material designated for cultivation experiments was washed twice with sterile artificial seawater and stored at 4°C, while material for DNA extraction (biofilms and filters) was stored at −20°C and transferred on dry ice to DSMZ, Braunschweig, Germany for further processing.

### Filtration of Water Samples

Triplicate water samples were homogenized by gentle stirring and 1 L was used for filtration. Water was filtered through a polycarbonate membrane filter (Ø 47 mm, Isopore, Merck) with a pore size of 0.22 μm to collect planktonic microorganisms in the seawater occurring in proximity to the sampled *P. oceanica* meadow. Filters were frozen and stored at −20°C until DNA extraction.

### Seagrass and Biofilm Preparation

Young and aged *P. oceanica* leaves (cf. sampling section) were gently rinsed several times with filter-sterilized artificial seawater (Corning bottle top filters, Sigma-Aldrich) to remove unattached bacteria. Leaves of young and aged seagrass were immediately fixed in 1.5% (v/v) formaldehyde. Fixed leaves were stored in the dark at 4°C. The remaining leaves were further processed. Biofilms were scraped off into sterile deionized water or sterile artificial seawater using sterile scalpels. Biofilms in deionized water were immediately frozen and stored on dry ice until DNA extraction. In contrast, biofilms stored in artificial seawater were kept in the dark at 4°C for subsequent cultivation experiments.

### Isolation and Cultivation

Isolation and cultivation of Planctomycetes was performed as previously described (Wiegand et al., 2020). In detail, initial cultivation was carried out on solidified NAGH ASW medium. NAGH ASW medium consisted of 250 mL/L artificial seawater (ASW), 20 mL/L mineral salt solution and 2.37 g/L HEPES. The pH was adjusted to 7.5. Artificial seawater consisted of 46.94 g/L NaCl, 7.84 g/L Na_2_SO_4_, 21.28 g/L MgCl_2_⋅6H_2_O, 2.86 g/L CaCl_2_⋅2H_2_O, 0.384 g/L NaHCO_3_, 1.384 g/L KCl, 0.192 g/L KBr, 0.052 g/L H_3_BO_3_, 0.08 g/L SrCl_2_⋅6H_2_O and 0.006 g/L NaF. Trace element solution consisted of 1.5 g/L Na-nitrilotriacetate, 500 mg/L MnSO_4_⋅H_2_O, 100 mg/L FeSO_4_⋅7 H_2_O, 100 mg/L Co(NO_3_)_2_⋅6H_2_O, 100 mg/L ZnCl_2_, 50 mg/L NiCl_2_⋅6H_2_O, 50 mg/L H_2_SeO_3_, 10 mg/L CuSO_4_⋅5H_2_O, 10 mg/L AlK(SO_4_)_2_⋅12H_2_O, 10 mg/L H_3_BO_3_, 10 mg/L NaMoO_4_⋅2H_2_O and Na_2_WO_4_⋅2H_2_O, modified from Karsten and Drake (1995). Mineral salt solution was composed of 10 g/L nitrilotriacetic acid (NTA), 29.70 g/L MgSO_4_⋅7H_2_O, 3.34 g/L CaCl_2_⋅2H_2_O, 12.67 mg/L Na_2_MoO_4_⋅2H_2_O, 99 mg/L FeSO_4_⋅7H_2_O and 50 mL/L metal salt sol. 44. Metal salt sol. 44 was composed of 250 mg/L Na-ethylenediaminetetraacetate (EDTA), 1.095 g/L ZnSO_4_⋅7H_2_O, 0.5 g/L FeSO_4_⋅7H_2_O, 154 mg/L MnSO_4_⋅H_2_O, 39.2 mg/L CuSO_4_⋅5H_2_O, 24.8 mg/L Co(NO_3_)_2_⋅6H_2_O and 17.7 mg/L Na_2_B_4_O_7_⋅10H_2_O. The vitamin solution was composed of 4 mg/L biotin, 4 mg/L folic acid, 20 mg/L pyridoxine-HCl, 10 mg/L riboflavin, 10 mg/L thiamine-HCl⋅2 H_2_O, 10 mg/L nicotinamide, 10 mg/L Ca-pantothenate, 0.2 mg/L vitamin B_12_ and 10 mg/L *p*-aminobenzoic acid. To solidify the medium, either 8 g/L gellan gum (initial cultivation) or 12 g/L agar (washed three times with deionized water; used for maintenance of isolated bacterial strains) were autoclaved separately and added to the medium prior to pouring plates. For initial cultivation, 20 mL/L of a 5% (w/v) solution *N*-acetyl-D-glucosamine (NAG) was added as sole carbon and nitrogen source and 20 mL/L nystatin suspension served as anti-fungal agent. Additionally, 500 mg/L streptomycin and 100 mg/L ampicillin were added to enrich Planctomycetes and suppress growth of other heterotrophic bacteria. For subsequent cultivation of novel isolates, medium M1H NAG ASW was prepared by additionally adding 0.25 g/L peptone, 0.25 g/L yeast extract, and 10 mL/L of a 2.5% (w/v) glucose solution.

Solid medium NAGH ASW was inoculated with 1:100 diluted biofilm suspensions of young and aged *P. oceanica* leaves. In addition, *P. oceanica* leaves were swabbed over NAGH ASW plates and pieces were placed on the plates. All inoculated cultures were incubated at 20°C in the dark until colony formation was visible. Parameters for colony selection were slow growth, a pink or cream pigmentation and a smooth colony surface. Selected colonies were subjected to several rounds of dilution plating. Isolated strains were identified by direct amplification and subsequent sequencing of the 16S rRNA gene using the optimized universal primers 8f (5′–AGA GTT TGA TCM TGG CTC AG–3′) and 1492r (5′–GGY TAC CTT GTT ACG ACT T–3′) modified from Lane (1991). PCR reactions were performed directly on single colonies, employing the following two-step protocol: step 1, initial denaturation at 94°C, 5 min, 10 cycles of denaturation at 94°C, 30 s, annealing at 59°C, 30 s, elongation at 72°C 1 min; step 2, 20 cycles denaturation at 94°C, 30 s, annealing at 54°C, 30 s, elongation at 72°C, 1 min and a final elongation step at 72°C, 7 min. All amplifications were carried out using an Applied Biosystems Veriti thermal cycler (Thermo Fisher Scientific) and PCR products were stored at 4°C until Sanger sequencing. Novelty of planctomycetal isolates was first checked by BLASTn analyses of 16S rRNA genes and isolates with sequence identity values below a 97% threshold were further investigated. The following additional primers were used for sequencing: 341f (5′-CCT ACG GGW GGC WGC AG-3′) (Muyzer et al., 1993), 515f (5′-GTG CCA GCA GCC GCG G-3′) (Lane, 1991), 515r (5′-CCG CGG CTG CTG GCA C-3′) (Muyzer et al., 1993), 1055f (5′-ATG GCT GTC GTC AGC T-3′) (Lee et al., 1993), and 1055r (5′-AGC TGA CGA CAG CCA T-3′) (Lee et al., 1993; Zhang et al., 2013). Sequences were cured manually and assembled employing the ContigExpress application of the Vector NTI Advance 10 software (Thermo Fisher Scientific) or the DNA Man tool (Lynnon Biosoft Corporation).

### Wide-Field Light Microscopy and Average Cell Size Determination

Bacterial cells were immobilized on a 1% (w/v) agarose-pad in MatTek 35 mm glass-bottom dishes and imaged under phase-contrast illumination using a Nikon Eclipse Ti inverse microscope at 100-fold magnification and employing the Nikon DS-Ri2 camera. To determine the cell size of the novel planctomycetal strains, 100 individual cells of each strain were measured using the NIS-Elements software V4.3 (Nikon Instruments).

### Field Emission Scanning Electron Microscopy (SEM) of Bacteria and Seagrass Leaf Biofilms

Cells were fixed in modified HEPES buffer (3 mM HEPES, 0.3 mM CaCl_2_, 0.3 mM MgCl_2_, 2.7 mM sucrose, pH 6.9) containing 1% (v/v) formaldehyde for 1 h on ice and were washed once with the same buffer. Cover slips with a diameter of 12 mm were coated with a poly-L-lysine solution (Sigma-Aldrich) for 10 min, washed with distilled water and air-dried. Seagrass leaves, fixed with 1.5% formaldehyde, or 50 μL of the fixed bacteria solution were placed on a cover slip and allowed to settle for 10 min. Cover slips were then fixed in TE buffer (20 mM TRIS, 1 mM EDTA, pH 6.9) containing 1% glutaraldehyde for 5 min at room temperature and subsequently washed twice with TE–buffer before dehydrating in a graded series of acetone (10, 30, 50, 70, 90, 100%) on ice for 10 min at each concentration. Samples from the 100% acetone step were brought to room temperature before placing them in fresh 100% acetone. Samples were then subjected to critical-point drying with liquid CO_2_ (CPD 300, Leica). Dried samples were covered with a gold/palladium (80/20) film by sputter coating (SCD 500, Bal-Tec) before examination in a field emission scanning electron microscope (Zeiss Merlin) using the Everhart Thornley HESE2–detector and the in-lens SE–detector in a 25:75 ratio at an acceleration voltage of 5 kV.

### Physiological Analyses

The planctomycetal isolate KOR34^T^ was grown in M1H NAG ASW medium to the early stationary phase and glass tubes were inoculated 1:10. Growth at temperatures of 10, 15, 20, 22, 24, 27, 30, 33, 36, 40°C was determined by OD_600_ measurement of triplicates. To determine the pH optimum, M1H NAG ASW medium was buffered to pH 5.0, 5.5, 6.0, 6.5, 7.0, 7.5, 8.0, 8.5, 9.0, and 9.5 at 100 mM final concentrations with MES, HEPES, HEPPS or CHES buffers. Due to flaky growth of the other isolate, KOR42^T^, it was grown in tubes containing M1H NAG ASW medium for 14 days and change in turbidity was captured by photography to determine the temperature and pH optimum (10°C was omitted since no growth was observed). Final determination of optimal growth conditions was achieved by analyzing resulting growth curves and calculating growth rates (Supplementary Figure S2). Catalase activity was determined by reaction of fresh cell material with 3% H_2_O_2_ solution, resulting in the release of oxygen (catalase-positive) or in no observable reaction (catalase-negative). Cytochrome oxidase activity was determined using Bactident Oxidase test stripes (Merck Millipore) following the manufacturer’s instructions. Substrate utilization of strains KOR34^T^ and KOR42^T^ was determined using the Biolog GN2 MicroLog test panel for Gram-negative bacteria. Sterile glass tubes were prepared in duplicates with a basic sterile medium mixture containing 14.2 mL IF-0a inoculation fluid (Biolog), 1.6 mL of a 10x salt solution (Buddruhs et al., 2013), 160 μL 1M HEPES buffer (pH 8.0), 80 μL double concentrated vitamin solution and 16 μL trace element solution. Tubes were inoculated with bacterial colony material to a turbidity of 50–60%. The cell suspensions were then used to inoculate substrate plates (Biolog GN2 MicroLog). To enable the comparison of substrate utilization values, the data of each single experiment were normalized to 100. Only values corresponding to >25% substrate utilization were considered positive. A heat map graphic was obtained in the R environment (R Core Team, 2015) by using the heatmap.2 function of the gplots package.

### Cellular Fatty Acid Analysis

Biomass of strains KOR34^T^ and KOR42^T^ was obtained from liquid cultures grown in M1H NAG ASW medium under optimal growth conditions until the stationary phase. 30 mg of lyophilized biomass was analyzed by the Identification Service of the German Collection of Microorganisms and Cell Cultures (DSMZ) according to the standard protocols of the facility (Miller, 1982; Kuykendall et al., 1988; Kämpfer and Kroppenstedt, 1996).

### Genome Data

Genome sequences of strains KOR34^T^ and KOR42^T^ are available from NCBI GenBank under accession numbers SIHJ00000000 (KOR34^T^) and SIHI00000000 (KOR42^T^) (Wiegand et al., 2020). 16S rRNA gene sequences are available from GenBank under accession numbers MK554542 (KOR34^T^) and MK554543 (KOR42^T^).

### Phylogenetic Analysis and Tree Reconstruction

16S rRNA gene sequence-based phylogeny was computed for strains KOR34^T^ and KOR42^T^, the type strains of all described planctomycetal species (assessed in January 2020) including the isolates recently published (Wiegand et al., 2020). The 16S rRNA gene sequences were aligned with SINA (Pruesse et al., 2012) and the phylogenetic inference was calculated with RAxML (Stamatakis, 2014) with a maximum likelihood approach with 1,000 bootstraps, nucleotide substitution model GTR, gamma distributed rate variation and estimation of proportion of invariable sites (GTRGAMMAI option). Three 16S rRNA genes of bacterial strains from the PVC superphylum outside of the phylum *Planctomycetes* were used as outgroup. The *rpoB* nucleotide sequences were taken from the above-mentioned as well as from other publicly available genome annotations and the sequence identities were determined as described (Bondoso et al., 2013). Upon extracting those parts of the sequence that would have been sequenced with the used primer set the alignment and matrix calculation was done with Clustal Omega (Sievers et al., 2011). The average nucleotide identity (ANI) was calculated using OrthoANI (Lee et al., 2016). The average amino acid identity (AAI) was calculated using the aai.rb script of the enveomics collection (Rodriguez-R and Konstantinidis, 2016) and percentage of conserved proteins (POCP) was calculated as described (Qin et al., 2014).

### Microbial Community Analysis

DNA from *P. oceanica* biofilms obtained from 10 leaves (five old- and five young leaves) along with DNA from water filters was extracted using the PowerBiofilm DNA Isolation Kit (MoBio Laboratories, Dianova) following the manufacturer’s protocol with a few exceptions: Time of incubation at 37°C in buffer B1 was increased to an overnight step. Incubation at 55°C was increased to 30 min. Incubation at 4°C was increased to 20 min. Bead-beating was performed in a FastPrep-24 instrument (MP Biomedicals) at 5.5 m/s for 30 s. DNA was eluted in 100 μL BF7 buffer and stored at −20°C until further processing. Genomic DNA extracted from water filters and seagrass biofilms was amplified with the illustra GenomiPhi V3 DNA Amplification Kit (GE Healthcare) (Dean et al., 2002) following the general recommendations of the manufacturer. For one single amplification reaction (20 μL total volume), 1 ng of genomic DNA was used. To reduce remaining stochastic amplification bias, three independent reactions per filter were pooled. Amplification reactions were performed in a thermal cycler (Veriti 96-Well, Applied Biosystems). Multiple displacement amplification (MDA) gDNA was stored at −20°C until further processing. Amplification of the variable region 3 (V3) of the 16S rRNA gene was performed using two subsequent PCR amplifications. The first protocol was used to enrich the V3 region of MDA gDNA obtained from water filters and plant biofilms. In this protocol, the universal forward primer 341f (5′-CCT ACG GGW GGC WGC AG-3′) and the reverse primer uni515r (5′-CCG CGG CTG CTG GCA C-3′) (modified from 518r) (Muyzer et al., 1993) were used. The second PCR protocol was then performed with extended V3 region primers V3F (5′-AAT GAT ACG GCG ACC ACC GAG ATC TAC ACT CTT TCC CTA CAC GCT CTT CCG ATC TCC TAC GGG WGG CWG CAG-3′) and indexed V3R primers (5′-CAA GCA GAA GAC GGC ATA CGA GAT NNN NNN GTG ACT GGA GTT CAG ACG TGT GCT CTT CCG ATC TCC GCG GCT GCT GGC AC-3′), modified from Bartram et al. (2011). The first PCR consisted of an initial denaturation step at 94°C, 5 min, followed by 10 cycles of denaturation at 94°C, 1 min, annealing at 63°C, 1 min, elongation at 72°C, 1 min and a final elongation step at 72°C, 10 min. Three independent pre-amplification reactions were pooled and stored at 4°C until further processing. In the second PCR, an initial denaturation step at 98°C, 5 min was followed by 10 cycles of denaturation at 98°C, 1 min, annealing at 65°C, 1 min, elongation at 72°C, 1 min and a final elongation step at 72°C for 5 min. To reduce stochastic amplification bias, three independent amplifications were performed.

The Amplicon library was sequenced on an Illumina MiSeq using V3 chemistry, 301 cycles per read and paired end settings. Raw sequences were subjected to adapter clipping and quality trimming using Trimmomatic v.0.36 (Bolger et al., 2014) with the following arguments: “LEADING:3 TRAILING:3 SLIDINGWINDOW:4:15 MINLEN:105.” Overlapping read pairs were identified and merged using FLASH v.1.2.11 (Magoč and Salzberg, 2011). Due to the short length of the amplicon inserts, amplicon read pairs overlapped in their entire sequence length. Therefore, merging resulted in high confidence consensus sequences which minimized the influence of random sequencing errors. Merged amplicon reads were furthermore analyzed and filtered based on the presence of the employed V3 region-specific forward and reverse primer sequences, which were subsequently clipped from the reads. A length filter for sequences between 120 and 167 base pairs was applied. Sequences below or above this cut-off, respectively, were found to be chimeric sequences that were not detected by the UCHIME algorithm. Taxonomic classification diversity analysis of the processed sequences was performed using QIIME 1 (Caporaso et al., 2010). Operational taxonomic units (OTUs) were generated with UCLUST, applying a 97% identity threshold (Edgar, 2010). Reference for OTU clustering and taxonomic classification was the SILVA database, version 123 (Quast et al., 2013; Yilmaz et al., 2014).

## Results and Discussion

### Biofilm Morphology and Bacterial Community Analyses

*P. oceanica* leaves from Corsican seagrass meadows were macroscopically distinct. While young leaves were smooth, showing little to no visible colonization, aged leaves were rough and heavily colonized by all sorts of organisms (Figure 1). Ignoring macroscopic epiphytes that were already addressed in detail before (Ben Brahim et al., 2010, 2014), we used scanning electron microscopy to focus on the microbial biofilm. We found that young leaves were mainly colonized by bacteria (Figures 2A,B), while aged leaves were additionally colonized by diatoms and other protists (Figures 2C,D). The colonization of *P. oceanica* with diatoms, including members of the genus *Cocconeis*, was previously reported (Novak, 1984; Mazzella and Spinoccia, 1992). Morphologically, the observed diatoms might very well belong to the genus *Cocconeis* (Figure 2C). Such species were previously described as early colonizers on *Zostera noltii* seagrass (Lebreton et al., 2009), while they appear to be later colonizers in our study when considering that they were almost exclusively found on aged leaves. At higher magnification (Figures 2B,D), morphological details of individual bacterial cells became visible and cells displaying typical planctomycetal characteristics were observed (Figures 2B,D, white arrowheads). Indicative for the presence of planctomycetal cells is the pear-shaped or ovoid cell morphology and polar budding as mode of cell division. These traits are common among planctomycetal species and make them easily distinguishable from other microorganisms (Wiegand et al., 2018). However, applying these criteria to determine the exact amount of Planctomycetes on *P. oceanica* leaves would fall short as some planctomycetal species divide by binary fission or display different cell shapes (Wiegand et al., 2018). Visual analysis of microbial cells on a larger SEM micrograph (Supplementary Figure S1) revealed that more than 40% of the bacterial community consists of cells resembling the typical planctomycetal morphology. This is among the highest percentages of Planctomycetes in microbial biofilms found so far (Bengtsson and Øvreås, 2010; Wiegand et al., 2018). Our results suggest that observed cells displaying a planctomycetal morphology appear to be major players in *P. oceanica* leaf biofilms. Furthermore, these cells frequently form microcolonies (Figures 2A,B), patches of cells of very similar shape that divide via polar budding. Occurrence of such microcolonies is surprising when taking into account that Planctomycetes divide rather slowly. When considering the growth disadvantage, a patchier distribution of Planctomycetes in natural biofilms should be expected since faster growing competitors would prevent planctomycetal microcolony formation.

For a deeper analysis of *P. oceanica* leaf-associated bacterial community compositions V3 amplicons of the 16S rRNA gene were constructed and sequenced. 22,887 and 26,429 sequences were obtained from young and aged leaves, respectively. To compare the *P. oceanica* leaf microbiome with the surrounding water, 28,118 sequences were gained from the filtered water sample. The numbers of operational taxonomic units (OTUs) of young and old leaves were 551 and 611, respectively. The surrounding seawater yielded 489 OTUs (Supplementary Table S1). While all three OTU values are roughly within the same range, a slight tendency toward higher species richness in biofilms versus the surrounding water becomes visible. This tendency is supported by calculating the alpha diversity as measure of biodiversity (species richness): aged leaf biofilms display the highest diversity, biofilms from young leaves are less rich in species and the surrounding water shows the lowest biodiversity. This points to the importance of *P. oceanica* meadows for bacterial diversity and thus stability of the ecosystem with marine surfaces as hotspots for bacterial interspecies interactions.

Classification of the datasets on phylum level revealed that biofilms of both, young and aged leaves, were dominated by bacteria of the phylum *Planctomycetes*. 85.4 and 83.2% of the obtained sequences were of planctomycetal origin (Figure 3). This is by far the highest abundance of Planctomycetes measured in a natural biofilm environment so far (Wiegand et al., 2018). Other major players in young and old leaf biofilms were the phyla *Proteobacteria* (12%/9%) and *Verrucomicrobia* (2%/5%). In contrast, the surrounding water was dominated by the phyla *Proteobacteria* (63%), *Cyanobacteria* (16%) and *Bacteroidetes* (16%), whereas *Planctomycetes* accounted only for 1.3% of the OTUs (Figure 3).

The highest abundance of Planctomycetes in marine biofilms reported thus far is 70% of the bacterial community on the macroalga *Ecklonia radiata* in Australia (Wiegand et al., 2018). The second highest abundance (56%) was found in biofilms of the macroalga *Laminaria hyperborea* (Bengtsson and Øvreås, 2010). Both values indicate that a natural abundance of Planctomycetes of more than 80% is imaginable. Furthermore, the experimental design (pooling of leaves from different plants as biofilm source and two independent experiments for biofilms on aged and young leaves) suggests that DNA extraction or amplification artifacts are unlikely to explain the measured values. Furthermore, our SEM micrographs show a high abundance (46%) of cells with a planctomycetal cell morphology. However, in case of biofilms from the macroalga *L. hyperborea*, seasonal dynamics, macrofouling and other factors strongly influence the bacterial community and members of the phylum *Planctomycetes* did not show high abundances at all times (Bengtsson et al., 2010; Sawall et al., 2012). In addition, analyses of marine macroalgal biofilms have shown that Planctomycetes are almost always present, albeit not always as the dominant fraction of the bacterial communities (Lachnit et al., 2011; Zhang et al., 2013). Metagenomic analysis of e.g., the giant kelp *Macrocystis pyrifera* revealed that the bacterial biofilm community is predominantly comprised of the phyla *Proteobacteria* and *Bacteroidetes*, with *Planctomycetes* as minor, but still substantial fraction of 4% (Vollmers et al., 2017). In conclusion, amplicon analysis demonstrated that the phylum *Planctomycetes* can account for more than 80% of the bacterial species in *P. oceanica* leaf biofilms. Despite the snapshot character of the study, Planctomycetes seem to be important players in this habitat.

Taken together, while the endophytic root microbiome and mycobiome has been investigated extensively (Garcias-Bonet et al., 2012; Torta et al., 2015; Vohnik et al., 2015, 2016), to the best of our knowledge, this is the first morphological and amplicon-based analysis of the *P. oceanica* leaf microbiome. The surprising result that Planctomycetes were the predominant colonizers of this habitat calls for future investigations with seasonal monitoring, not only of biofilms from shallow waters, but also deep-water seagrass meadows.

### Isolation and Characterization of Novel Planctomycetal Strains From the *P. oceanica* Leaf Microbiome

#### Isolation of the Strains

Basic isolation and characterization aspects of *P. oceanica*-associated Planctomycetes have been described as part of a comprehensive study covering all sorts of habitats and yielding more than 70 novel planctomycetal species (Wiegand et al., 2020). We followed a stringent cultivation approach with *N*-acetyl-D-glucosamine as sole carbon and nitrogen source, combined with the use of an antibiotic mixture and gellan gum as solidifying agent in order to selectively enrich planctomycetal bacteria from young and aged *P. oceanica* leaves. Swabbing of seagrass leaves over petri dishes with solid medium turned out to be most efficient and yielded first colonies visible after 7 days and more after 4 months of incubation. In total, 85 colonies met our screening criteria and were further investigated. 85% of colonies obtained belong to the phylum *Planctomycetes*, demonstrating the efficiency of the enrichment process. The other 15% of screened colonies were mostly related to *Sphingopyxis* and *Erythrobacter*. Interestingly, such strains originated from colonies that were harvested after a minimum of 20 days of incubation, indicating that the time window for future cultivation attempts targeting Planctomycetes from comparable habitats might be best focused on colonies that appear after 1–4 months. Among the obtained Planctomycetes, the genera *Blastopirellula* (22% on young and 27% on aged seagrass leaves) and *Rhodopirellula* (9% on young and 25% on aged leaves) were most frequent. However, two strains, KOR34^T^ and KOR42^T^, were identified to be phylogenetically distinct from all validly described Planctomycetes and were therefore chosen for detailed analysis. Both strains were isolated from aged seagrass leaves.

#### Phylogenetic Inference

**FIGURE 4:**
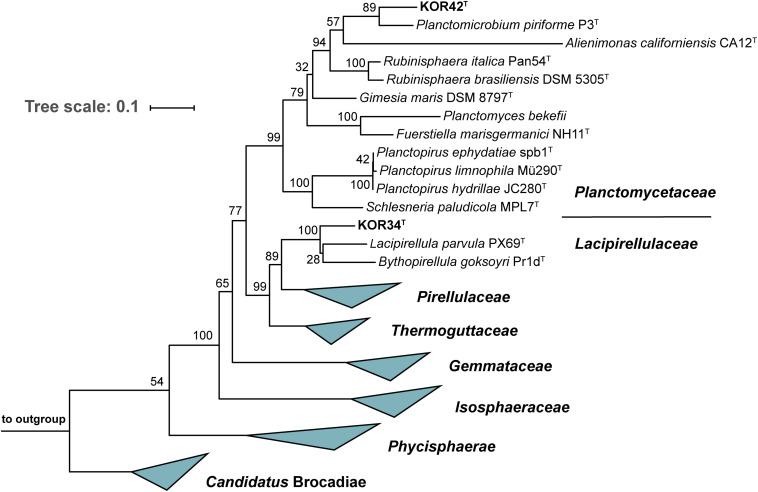
Maximum likelihood 16S rRNA gene-based phylogenetic analysis. The phylogenetic tree was constructed as described in the “Materials and Methods” section. Bootstrap values obtained after 1,000 re-samplings for each node are given in %. Three 16S rRNA genes from the PVC superphylum outside the phylum *Planctomycetes* served as outgroup.

To determine the precise phylogenetic position of the two novel strains, phylogenetic tree reconstruction was performed (Figure 4). Both strains cluster within the class *Planctomycetia*, KOR34^T^ in the family *Lacipirellulaceae* and strain KOR42^T^ in the family *Planctomycetaceae*. The closest neighbors of KOR34^T^ turned out to be *Bythopirellula goksoyri* Pr1D^T^ and the recently described *Lacipirellula parvula* PX69^T^ (both 91.6% 16S rRNA gene similarity to KOR34^T^) (Storesund and Øvreås, 2013; Dedysh et al., 2020). Strain KOR42^T^ is closely related to *Planctomicrobium piriforme* P3^T^ (92.2% 16S rRNA gene similarity) (Kulichevskaya et al., 2015). Distinctly lower identity values were found during comparison of the strains to type species of other related genera (Supplementary Table S2). Minimal 16S rRNA gene identity thresholds for genera of 94.5% (Yarza et al., 2014) suggest that both strains belong to novel genera. This assumption is supported by *rpoB* sequence identity values (Supplementary Table S2) below the upper value of 78% of the proposed genus threshold range (Bondoso et al., 2013; Kallscheuer et al., 2019). Further support is provided by values of AAI and POCP. The genus thresholds for these phylogenetic markers are 60–80% (Luo et al., 2014) and 50% (Qin et al., 2014), respectively and all determined values for strains KOR34^T^ and KOR42^T^ are clearly below these thresholds (Supplementary Table S2).

#### Morphological Analysis

**FIGURE 5:**
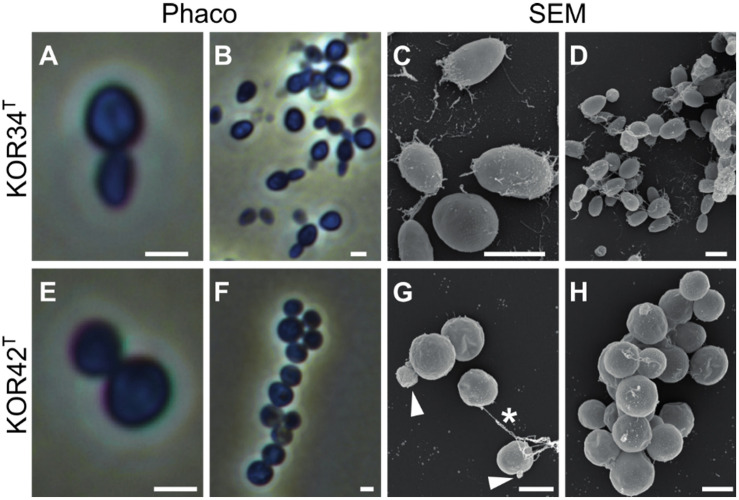
Microscopic analysis of the cell morphology of strains KOR34^T^ and KOR42^T^. Morphology of strains KOR34^T^ and KOR42^T^ observed with phase-contrast (Phaco) and scanning electron microscopy (SEM). Cells of KOR34^T^ are ovoid to pear-shaped with one pole bigger than the other **(A,C)**. Cells are connected and form rosettes or larger aggregates **(B,D)**. Cells of KOR42^T^ are of spherical morphology **(E,H)** and form chains and aggregates **(F,G)**. Cells divide by budding (**G**, white arrowheads) and are connected (**G**, white asterisk). Scale bar is 1 μm.

Strains KOR34^T^ and KOR42^T^ both form cream-colored colonies with a smooth surface. KOR34^T^ cells are ovoid to pear-shaped (Figures 5A–D), divide by polar budding (Figure 5A) and form multicellular rosettes and aggregates (Figures 5B,D). The average cell size is 1.4 × 1.1 μm (Table 1). KOR42^T^ cells are of spherical shape (Figures 5E–H) and also divide by polar budding (Figure 5E). Multicellular rosettes, filaments of single cells and aggregates were observed (Figure 5F). This type of filament formation is rather unique for Planctomycetes and was previously only described for *Isosphaera pallida* (Giovannoni et al., 1987). However, KOR42^T^ forms such filaments only occasionally, while *I. pallida* always forms filaments (Giovannoni et al., 1987). The average cell diameter of KOR42^T^ is 1.7 ± 0.2 μm (Table 1). Cells with the morphotypes of both strains were visible in SEM micrographs of *P. oceanica* leaf biofilms (Figure 2).

**TABLE 1:** Phenotypic characteristic of the two novel planctomycetal isolates.

Characteristic	KOR34^T^	KOR42^T^
Arrangement of cells	Rosettes and aggregates	Rosettes, chains and aggregates
Cell size [μm]	1.4 ± 0.2 × 1.1 ± 0.2	1.7 ± 0.2
Cell shape	Ovoid to pear-shaped	spherical
Source of isolation	*Posidonia oceanica* biofilm	*Posidonia oceanica* biofilm
Colony color	Cream	Cream
Relation to oxygen	Aerobic	Aerobic
Oxidase activity	+	+
Catalase activity	+	+
Temperature range [°C]	16–36	22–36
Temperature optimum [°C]	33	33
pH range	6.0 – 8.5	5.5 – 8.5
pH optimum	7.0	7.0 -7.5
Major fatty acid [%]	C_18__:__1_ ω9c (45.8)	C_16__:__0_ (42.3)

### Physiological Analysis and Genomic Parameters

Strain KOR34^T^ was able to grow between 15-36°C with an optimum at 33°C (Supplementary Figure S2A). A pH between 6.0 and 8.5 was tolerated with optimal growth at pH 7.0 (Supplementary Figure S2B). Fatty acid analysis revealed C_18__:__1_ ω9c as the major component, accounting for 45.8% of fatty acids detected.

Strain KOR42^T^ grew between 22 and 36°C with an optimum at 33°C. For survival, strain KOR42^T^ required a larger air-filled headspace during cultivation compared to other aerobic Planctomycetes that we recently obtained (Wiegand et al., 2020). In addition, strain KOR42^T^ grows in flake-like aggregates when incubated in M1H NAG ASW medium at temperatures from 22 to 27°C (Supplementary Figure S3A). However, a homogeneous culture was observed at temperatures of 30, 33, or 36°C (Supplementary Figure S3A). Both, aggregated and homogeneous cultures contained intact cells, which divide by budding. Known aggregate-inducing effectors such as increase of oxygen concentration (Sigalevich et al., 2000), addition of autoinducer 2 (Laganenka et al., 2016) or stress-inducing conditions (Hall-Stoodley et al., 2004; Klebensberger et al., 2007) were not further analyzed in our study. Strain KOR42^T^ tolerates pH values between 6.5 and 8.5 with an optimum at pH 7.5 (Supplementary Figure S3B). Fatty acid analysis revealed C_16__:__0_ as the major compound, accounting for 42.3% of fatty acids detected (Table 1).

Both strains, KOR34^T^ and KOR42^T^ turned out to be cytochrome oxidase and catalase positive when grown on solid medium. They utilized a variety of carbon sources including dextrin (only KOR34^T^), glycogen (only KOR34^T^), *N*-acetyl-galactosamine, *N*-acetyl-glucosamine, arabinose, cellobiose, fructose, fucose, galactose, gentiobiose, lactose, lactulose, maltose, mannose, mellibiose, β-methyl-glycoside, rhamnose, sucrose, trehalose, turanose, pyruvic acid methyl ester (only KOR34^T^), succinic acid mono-methyl-ester, galacturonic acid (only KOR34^T^), glucuronic acid (only KOR34^T^), lactic acid (only KOR34^T^), glutamic acid (only KOR34^T^), glucuronamide (only KOR42^T^) and glycerol (only KOR34^T^). The complete carbon utilization patterns are summarized in Supplementary Figure S4. Next to the sugars listed above, glucose, galactose, and mannose are utilized by both strains. These are among the main sugars of *Posidonia australica* (Torbatinejad et al., 2007). In addition, like terrestrial plants, *Posidonia* contains high proportions of cellulose, up to 200 g/kg dry mass. This is in line with the capability of both isolates to utilize cellobiose, a disaccharide composed of two β-1,4-linked glucose molecules, that can be obtained by hydrolysis of cellulose. Cellulolytic activities were reported for the Planctomycete *Telmatocola sphagniphila* (Kulichevskaya et al., 2012) and peat-inhabiting Planctomycetes possibly involved in the degradation of *Sphagnum*-derived litter (Ivanova et al., 2016), which is mainly constituted of cellulose (Kremer et al., 2004). However, it remains unclear if utilization of cellobiose is also indicative for the capability of the two strains to degrade cellulose itself.

The 6,765,537 bp genome of strain KOR34^T^ contains 5,344 genes of which 5,247 are putatively protein-coding. The molar G + C content is 66.7 ± 1.2% (Table 2). The 6,734,412 bp genome of strain KOR42^T^ contains 5,584 genes of which 5,508 are putatively protein-coding. The molar G + C content is 52.8 ± 1.8% (Table 2). These values are close to the average values obtained for planctomycetal genomes (Wiegand et al., 2020) and in a similar range as in the closest relatives *B. goksoyri* Pr1d^T^, *L. parvula* PX69^T^ and *P. piriforme* P3^T^ (Table 2).

**TABLE 2:** Genomic characteristics of strains KOR34^T^ and KOR42^T^.

	**KOR34^T^**	***L. parvula* PX69^T^**	***B. goksoyri* Pr1d^T^**	**KOR42^T^**	***P. piriforme* P3^T^**
Total genes	5,344	5,665	5,395	5,584	5,117
Genes/Mb	790	818	833	829	810
Giant genes	2	1	0	0	1
Protein-coding genes	5,247	5,581	5,306	5,508	5,050
Protein-coding genes/Mb	776	806	820	818	799
Hypothetical proteins	2,219	3,702	2,322	2,516	2,814
tRNAs	91	73	68	70	53
16S rRNA genes	1	1	1	1	1
Genome size (bp)	6,765,537	6,922,258	6,473,141	6,734,412	6,317,004
Coding density	86.5	84.7	86.5	85.7	85.8
Completeness	96.6	96.6	96.6	96.6	95.7
Contamination	0.0	3.5	1.7	0.0	1.7
# scaffolds	13	1	1	152	41
# contigs	19	1	8	162	41
G + C (%)	66.7 ± 1.2	61.7	52.8	52.8 ± 1.8	58.8 ± 1.7

### Potential for Production of Secondary Metabolites

Given the high abundance of Planctomycetes in the bacterial community of *P. oceanica* leaf biofilms, our hypothesis of planctomycetal secondary metabolite production to dominate competitive habitats appears tempting to address. How should a slow growing strain such as KOR34^T^ (maximal growth rate 0.039 h^–1^, generation time of 18 h) compete against faster growing bacteria such as members of the ‘*Roseobacter* clade’ (maximal growth rate of 0.43 h^–1^, generation time of 1.6 h) (Martens et al., 2006; Frank et al., 2014)? For addressing this question, we revisited the results of the antiSMASH analyses of the genomes of the two strains, which provided evidence for planctomycetal small molecule production in the past (see Wiegand et al., 2020 for details on methods). The genome of strain KOR34^T^ harbors six gene clusters, which encode enzymes putatively involved in the biosynthesis of secondary metabolites (Supplementary Table S3). Three of them appear to be related to the formation of terpenoids. The other three clusters are putatively involved in the formation of polyketides, non-ribosomal peptides or other amino acid-derived compounds. Strain KOR42^T^ harbors altogether five putative clusters in its genome, four of which appear to be involved in terpenoid biosynthesis (Supplementary Table S3). The remaining cluster harbors a gene coding for a putative type III polyketide synthase. One or more of these genes might be involved in the production of bioactive small molecules with potential anti-microbial activity. Biosynthesis of such compounds might allow for compensation of slower growth of the two isolated strains as soon as growth of competing microorganisms is also inhibited. In this context, planctomycetal genomes were found to be particularly rich in giant genes (Kohn et al., 2016, 2019). Such genes could be involved in an entirely novel way of small molecule assembly (Wiegand et al., 2020) apparently unpredictable by manual analysis or antiSMASH prediction. Further analysis beyond the scope of this study is thus required to study planctomycetal interactions with other bacteria on the level of the (secondary) metabolome in aquatic habitats dominated by Planctomycetes.

## Conclusion

In this study, we employed electron microscopy and amplicon sequencing focusing on the cultivation-independent analysis of the bacterial community composition of *P. oceanica* leaves. We identified *Planctomycetes* as major bacterial fraction on this biotic surface (>80%), while the surrounding seawater was dominated by *Proteobacteria* and *Bacteroidetes*. In addition, we obtained two novel strains of the phylum *Planctomycetes* in axenic culture and described both in a polyphasic approach, including genome analysis. Since our findings only portrait a snapshot of the epiphytic bacterial community of *P. oceanica*, it would be interesting to monitor this community more frequently to understand its potential role in *P. oceania* meadow development. The extremely high abundance of Planctomycetes provides additional evidence for their ability to thrive and dominate in nutrient-rich hotspots, potentially by outcompeting other microorganisms.

Based on the presented results, we conclude that strains KOR34^T^ and KOR42^T^ represent two novel genera and species within the phylum *Planctomycetes*, class *Planctomycetia*, order *Pirellullales* (KOR34^T^)/order *Planctomycetales* (KOR42^T^).

### Description of *Posidoniimonas* gen. nov.

*Posidoniimonas* (Po.si.do.ni.i.mo’nas. N.L. fem. n. *Posidonia* scientific genus name of Neptune grass; L. fem. n. *monas* a unit, monad; N.L. fem. n. *Posidoniimonas*, a bacterial unit isolated from the Neptune grass *Posidonia oceanica*).

Cells are ovoid to pear-shaped and form multicellular rosettes and aggregates. No chain or spore formation was observed. During exponential growth, most cells are highly motile. Cells have a Gram-negative cell envelope architecture and divide by polar budding. Organisms display mesophilic growth properties. Members belong to the phylum *Planctomycetes*, class *Planctomycetia*, order *Pirellulales*, family *Lacipirellulaceae*. The type species of the genus is *Posidoniimonas corsicana*.

### Description of *Posidoniimonas corsicana* sp. nov.

*Posidoniimonas corsicana* (cor.si.ca’na. L. fem. adj. *corsicana* Corsican; corresponding to the origin of the strain from the Mediterranean island Corsica).

Exhibits the following properties in addition to those given for the genus. Strain KOR34^T^ grows aerobically, colonies are cream-colored and have a smooth surface. Cells are 1.4 ± 0.2 × 1.1 ±0.2 μm in size and display cytochrome oxidase and catalase activity. Growth of the type strain was observed at temperatures between 15 and 36°C with an optimum at 33°C. Optimal pH is 7.0, with a tolerance from 6.0 to 8.5. Utilizes a variety of sugar substrates and carboxylic acids and grows solely with *N*-acetyl-D-glucosamine. Prefers gellan gum over agar as solidifier in media. The G + C content of the DNA is 66.7%. The type strain KOR34^T^ (DSM 104303^T^ = LMG 31362^T^) was isolated from the biofilm community of *Posidonia oceanica*.

### Description of *Thalassoglobus* gen. nov.

*Thalassoglobus* (Tha.las.so.glo’bus. Gr. fem. n. *thalassa* the sea; L. masc. n. *globus* sphere; N.L. masc. n. *Thalassoglobus* a sphere from the sea).

Cells are spherical and form rosettes and aggregates. Chain formation, but neither spore formation nor motility was observed. Cells have a Gram-negative cell envelope architecture, divide by budding and members display mesophilic growth properties. The predominant cellular fatty acid of the type species is C_16__:__0_. Members belong to the phylum *Planctomycetes*, class *Planctomycetia*, order *Planctomycetales*, family *Planctomycetaceae*. The type species of the genus is *Thalassoglobus neptunius*.

### Description of *Thalassoglobus neptunius* sp. nov.

*Thalassoglobus neptunius* (nep.tu’ni.us. L. masc. adj. *neptunius* of Neptune; corresponding to the origin of the strain from the Neptune grass *Posidonia oceanica*)

Exhibits the following properties in addition to those given for the genus. Grows aerobically while colonies are cream-colored and have a smooth surface. Cells are 1.7 ± 0.2 μm in size and display cytochrome oxidase and catalase activity. Growth of the type strain at temperatures between 22 and 36°C with an optimum at 33°C was observed. Optimum pH is between 7.0 and 7.5, with a tolerance from 5.5 to 8.5. Utilizes a variety of sugar substrates and carboxylic acids and grows solely with *N*-acetyl-D-glucosamine. Prefers gellan gum over agar as solidifier in media. The G + C content of the DNA is 52.8 mol%. The type strain KOR42^T^ (DSM 104081^T^ = LMG 29823^T^) was isolated from the epiphytic biofilm community of *Posidonia oceanica*.

## Data Availability Statement

The datasets generated for this study can be found in the NCBI Genbank. The accession numbers can be found in the article.

## Author Contributions

PR, OJ, and CB were involved in sampling. PR and TK performed most of the experiments and wrote a manuscript draft together with NK. SW performed genome sequencing and phylogenetic analysis. NK analyzed the putative secondary metabolite clusters. MSJ contributed to text preparation. CB and MR performed microscopic analyses. JV performed amplicon sequencing. MJ supervised PR and cultivated the strains. A-KK supervised JV and helped with sequencing of amplicons and genomes. CJ supervised the study, led the sampling expedition, and contributed to text preparation. All authors contributed to the article and approved the submitted version.

## Conflict of Interest

The authors declare that the research was conducted in the absence of any commercial or financial relationships that could be construed as a potential conflict of interest. The reviewer C-EW declared a shared affiliation, with no collaboration, with two of the authors, CJ and MJ, to the handling editor at time of review.

## Supplementary Material

FIGURE S1 Scanning electron micrograph of a young *P. oceanica* leaf biofilm. Microcolonies of cells with planctomycetal morphology (white circles) are spread over the biofilm. Scale bar is 6 μm.

FIGURE S2 Temperature **(A)** and pH optimum **(B)** of strain KOR34^T^. The optical density was measured at 600 nm and growth rates were calculated and plotted against the corresponding pH or temperature. Data represent average values from three biological replicates.

FIGURE S3 Temperature **(A)** and pH optimum **(B)** of strain KOR42^T^. Measurement of the optical density was not possible due to extreme aggregate formation of the strain. Instead, growth was analyzed visually.

FIGURE S4 Substrate utilization patterns of KOR34^T^ and KOR42^T^. Heat map of substrate utilization patterns of strains KOR34^T^ and KOR42^T^. Two biological replicates were performed using the MicroLog GN2 substrate plates. Both strains utilized a variety of sugar substrates and KOR34^T^ utilized a few sugar acids not converted by KOR42^T^. Color scale shows substrate usage in percent utilization. Values < 25% were below threshold and evaluated as negative.

TABLE S1 Number of sequences, operating taxonomic units (OTUs) and diversity indices (Chao1, Shannon and Simpson) of V3 16S rRNA gene amplicons.

TABLE S2 Similarity values for the novel isolates KOR34^T^ and KOR42^T^ compared to closely related species. The similarity values are given for each strain compared to the novel isolates. All values are given in %.

TABLE S3 Gene clusters putatively involved in the biosynthesis of secondary metabolites in KOR34^T^ and KOR42^T^ predicted by antiSMASH.
