# Supplementary material for: The Microbiome of Posidonia oceanica Seagrass Leaves Can Be Dominated by Planctomycetes
Source: Front Microbiol. 2020 Jul 10;11:1458. doi: 10.3389/fmicb.2020.01458 (PMC7366357; doi:10.3389/fmicb.2020.01458)
Supplement: FIGURE S1 — Scanning electron micrograph of a young P. oceanica leaf biofilm. Microcolonies of cells with planctomycetal morphology (white circles) are spread over the biofilm. Scale bar is 6 μm. [file Data_Sheet_1.pdf]

Supplementary Material

The Microbiome of *Posidonia oceanica* Seagrass Leaves Can Be Dominated by Planctomycetes

Timo Kohn[1†], Patrick Rast[2†], Nicolai Kallscheuer[1], Sandra Wiegand[3], Christian Boedeker[2], Mike S. M. Jetten[1], Olga Jeske[1,2], John Vollmers[3], Anne-Kristin Kaster[3], Manfred Rohde[4], Mareike Jogler[5] and Christian Jogler[1,5]*

[1] Department of Microbiology, Radboud University, Nijmegen, Netherlands,
[2] Leibniz-Institut Deutsche Sammlung von Mikroorganismen und Zellkulturen, Braunschweig, Germany,
[3] Institute for Biological Interfaces 5, Karlsruhe Institute of Technology, Eggenstein-Leopoldshafen, Germany,
[4] Central Facility for Microscopy, Helmholtz Centre for Infection Research, Braunschweig, Germany,
[5] Department of Microbial Interactions, Institute of Microbiology, Friedrich Schiller University, Jena, Germany

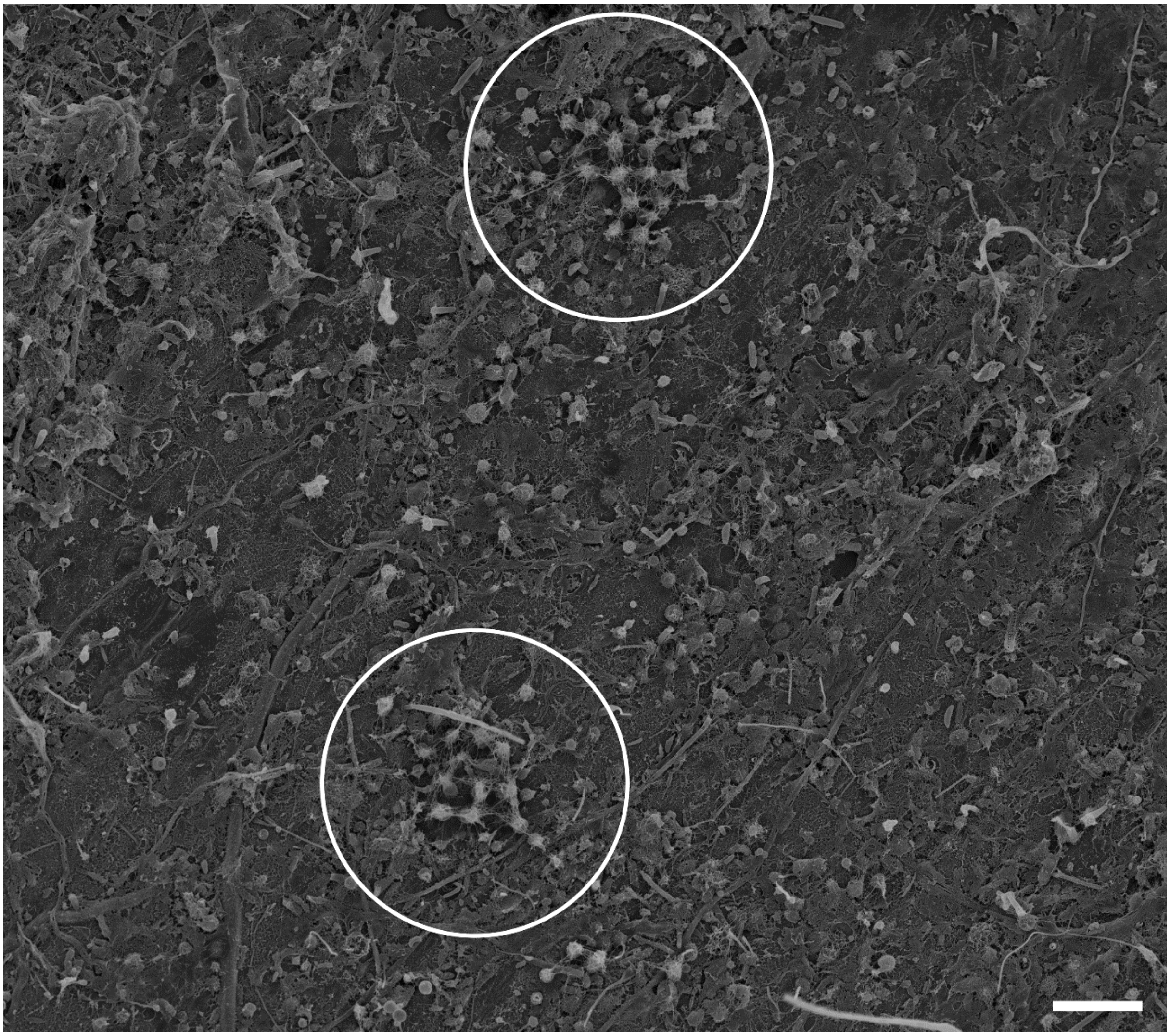

Figure S1. Scanning electron micrograph of a young *P. oceanica* leaf biofilm.

Microcolonies of cells with planctomycetal morphology (white circles) are spread over the biofilm. Scale bar is 6 µm.

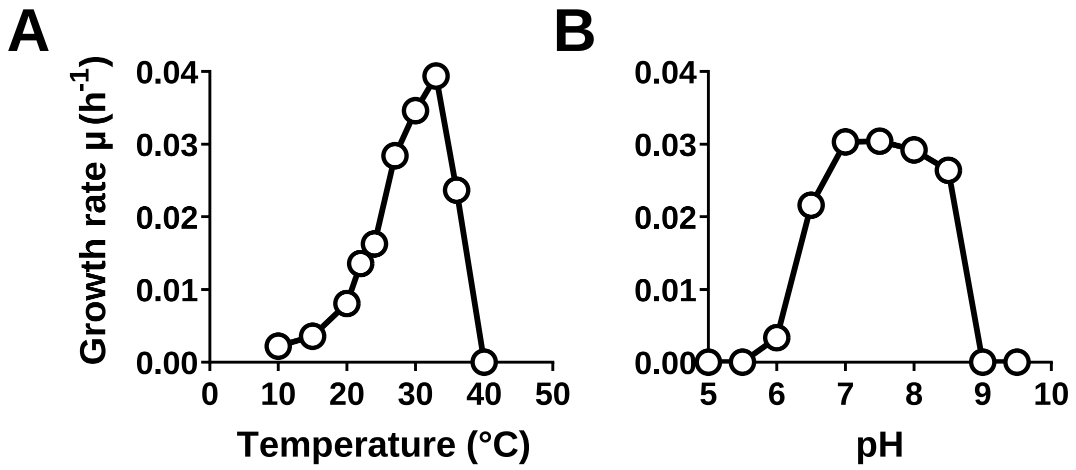

Figure S2. Temperature (A) and pH optimum (B) of strain KOR34^T.

The optical density was measured at 600 nm and growth rates were calculated and plotted against the corresponding pH or temperature. Data represent average values from three biological replicates.

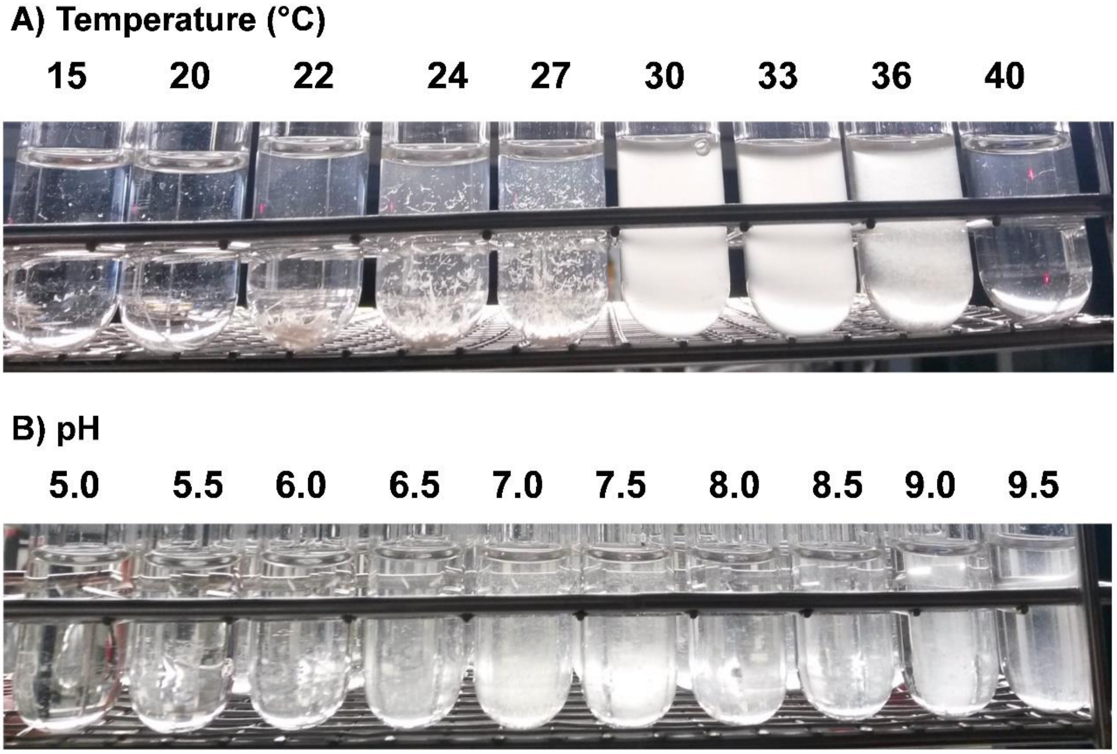

Figure S3. Temperature (A) and pH optimum (B) of strain KOR42^T.

Measurement of the optical density was not possible due to extreme aggregate formation of the strain. Instead, growth was analyzed visually.

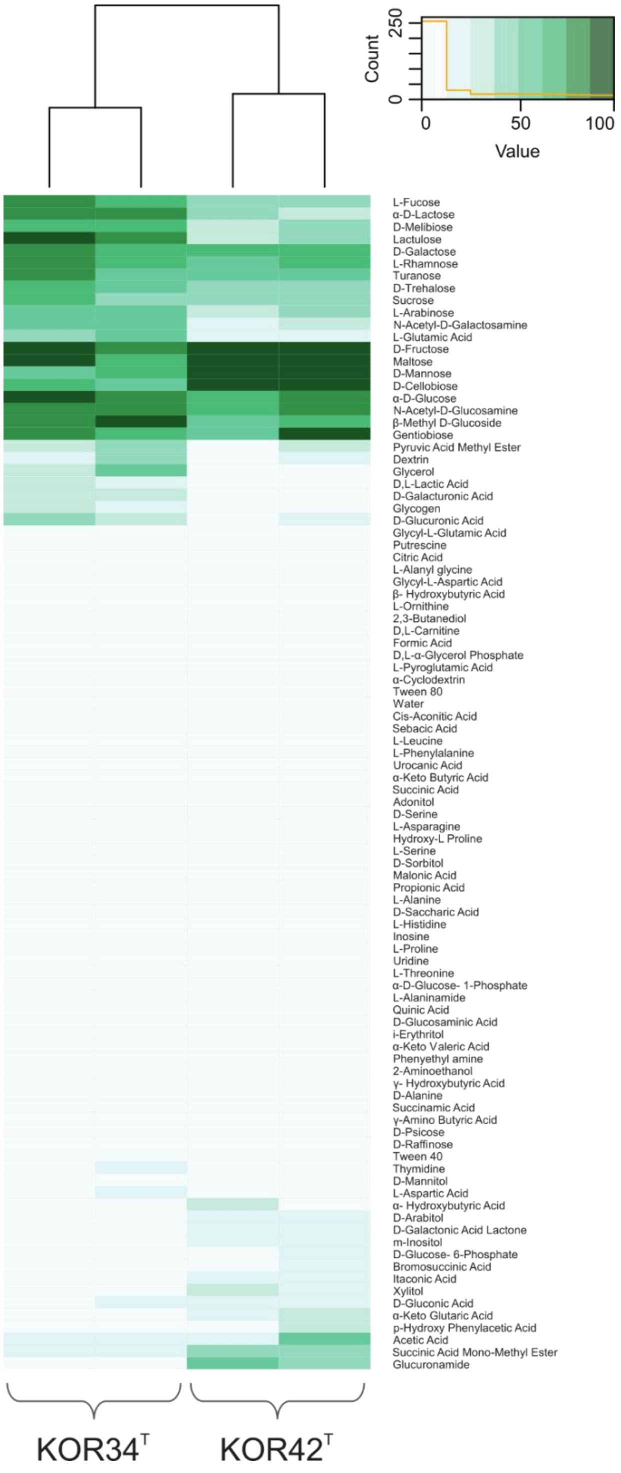

Figure S4. Substrate utilization patterns of KOR34^T and KOR42^T

Heat map of substrate utilization patterns of strains KOR34^T and KOR42^T. Two biological replicates were performed using the MicroLog GN2 substrate plates. Both strains utilized a variety of sugar substrates and KOR34^T utilized a few sugar acids not converted by KOR42^T. Color scale shows substrate usage in percent utilization. Values <25% were below threshold and evaluated as negative.

Table S1. Number of sequences, operating taxonomic units (OTUs) and diversity indices (Chao1, Shannon and Simpson) of V3 16S rRNA gene amplicons.

Sample	Sequences	OTUs	chao1	Shannon	Simpson	Planctomycetes
**Biofilm (young leaves)**	22,887	551	583.56	5.9	0.95	85.4 %
**Biofilm (old leaves)**	26,429	611	645.61	6.45	0.97	83.2 %
**Water sample**	28,118	489	508	5.36	0.94	1.3 %

Table S2. Similarity values for the novel isolates KOR34^T and KOR42^T compared to closely related species. The similarity values are given for each strain compared to the novel isolates. All values are given in %.

Strain	16S rRNA gene	*rpoB*	AAI	POCP
**KOR34^T**	100	100	100	100
***Bythopirellula goksoyri* Pr1d ^{T}**	91.6	75.5	55.4	45.9
***Lacipirellula parvula* PX69^T**	91.6	82.3	54.4	43.2
***Blastopirellula marina* DSM 3645^T**	88.1	71.3	50.2	35.6
***Stieleria maiorica* Mal15^T**	87	74.0	48.2	31.0
***Mariniblastus fucicola* FC18^T**	86.2	71.6	47.3	29.5
***Rhodopirellula baltica* SH1^T**	86.1	69.5	48.2	31.8
***Roseimaritima ulvae* UC8^T**	86	71.8	48.9	34.1
***Rhodopirellula rosea* LHWP3^T**	85.9	not available	not available	not available
***Rubripirellula obstinata* LF1^T**	84.9	67.6	47.7	27.4
***Pirellula staleyi* DSM 6068^T**	84.3	73.7	49.8	34.3
**KOR42^T**	100	100	100	100
***Planctomicrobium piriforme* P3^T**	92.2	76.4	57.8	47.1
***Rubinisphaera brasiliensis* DSM 5305^T**	88.7	71.5	52.2	42.3
***Gimesia maris* DSM 8797^T**	87.4	68.9	51.8	41.1
***Fuerstiella marisgermanici* NH11^T**	84.9	68.1	51.3	38.1
***Planctopirus limnophila* DSM 3776^T**	84.5	69.6	49.4	36.1
***Schlesneria paludicola* DSM 18645^T**	83.7	69.8	49.9	34.3
***Alienimonas californiensis* CA12^T**	81.8	67.7	49.0	34.9

Table S3. Gene clusters putatively involved in the biosynthesis of secondary metabolites in KOR34^T and KOR42^T predicted by antiSMASH.

**Locus tag**	**Putative annotation**
**KOR34^T**	
cluster 1 - polyketide	
**KOR34_06730**	type III polyketide synthase
cluster 2 - terpenoid	
**KOR34_14530**	ferredoxin family protein
**KOR34_14540**	radical SAM protein
**KOR34_14550**	polyprenyl synthetase
**KOR34_14560**	prenyltransferase and squalene oxidase repeat protein
**KOR34_14570**	squalene--hopene cyclase
cluster 3 - terpenoid	
**KOR34_18720**	pentalenene oxygenase
cluster 4 - mixed polyketide/ nonribosomal peptide	
**KOR34_27310**	nonribosomal peptide synthetase
**KOR34_27320**	oxidoreductase
**KOR34_27330**	nonribosomal peptide synthetase
cluster 5 - amino acid-derived compound	
**KOR34_39200**	aminotransferase class I/II
cluster 6 - terpenoid	
**KOR34_50450**	oxidoreductase
**KOR34_50460**	hypothetical protein
**KOR34_50470**	squalene--hopene cyclase
**KOR42^T**	
cluster 1 - terpenoid	
**KOR42_04850**	squalene synthase
cluster 2 - terpenoid	
**KOR42_08130**	squalene--hopene cyclase
cluster 3 - terpenoid	
**KOR42_18540**	squalene--hopene cyclase
**KOR42_18550**	polyprenyl synthetase
cluster 4 - polyketide	
**KOR42_21030**	type III polyketide synthase
cluster 5 - terpenoid	
**KOR42_26350**	squalene/phytoene synthase family protein
